# Contribution of sensory nerves to cutaneous reactive hyperaemia in non‐Hispanic Black and White young adults

**DOI:** 10.1113/EP091178

**Published:** 2023-04-08

**Authors:** Casey G. Turner, Demetria C. Walker, Brett J. Wong

**Affiliations:** ^1^ Department of Kinesiology & Health Georgia State University Atlanta Georgia USA; ^2^ Molecular Cardiology Research Institute Tufts Medical Center Boston Massachusetts USA

**Keywords:** arterial occlusion, human, microvascular function

## Abstract

The aim of this study was to assess cutaneous sensory nerve function, independent of nitric oxide, in non‐Hispanic Black and White young adults. We tested the hypothesis that cutaneous reactive hyperaemia and sensory nerve‐mediated vasodilatation would be lower in non‐Hispanic Black young adults relative to non‐Hispanic White young adults. Twenty‐four participants who self‐identified as non‐Hispanic Black (*n* = 12) or non‐Hispanic White (*n* = 12) were recruited. All participants underwent three bouts of reactive hyperaemia. An index of skin blood flow was measured continuously using laser‐Doppler flowmetry at a control site and at a site treated with topical 4% lignocaine to inhibit sensory nerve function. Peak reactive hyperaemia was lower in non‐Hispanic Black relative to non‐Hispanic White participants (*P* < 0.001). Total reactive hyperaemia was lower in non‐Hispanic Black [mean (SD); control, 4085 (955)%CVC_max_ s; lignocaine, 2127 (639) percent maximal cutaneous vascular conductance * seconds, %CVC_max_ s] relative to non‐Hispanic White [control: 6820 (1179)%CVC_max_ s; lignocaine, 3573 (712)%CVC_max_ s] participants (*P* < 0.001 for both sites). There was no difference between groups for the calculated contribution of sensory nerves to either the peak [non‐Hispanic Black, 25 (14)%; non‐Hispanic White, 19 (13)%] or total reactive hyperaemic response [non‐Hispanic Black, 48 (10)%; non‐Hispanic White, 47 (10)%]. These data suggest that cutaneous reactive hyperaemia is lower in non‐Hispanic Black young adults, but the sensory nerve contribution is similar in non‐Hispanic Black and White young adults.

## Abstract (highlights)

- **What is the central question of this study?**
  Does cutaneous sensory nerve‐mediated vasodilatation differ between non‐Hispanic Black and White young adults?
- **What is the main finding and its importance?**
  The magnitude of cutaneous reactive hyperaemia is lower in non‐Hispanic Black relative to non‐Hispanic White young adults, but the overall sensory nerve contribution is the same, suggesting that sensory nerve function is similar in both non‐Hispanic Black and White young adults.

## 1 INTRODUCTION

Non‐Hispanic Black individuals are more likely to develop cardiovascular and metabolic diseases, such as hypertension (Al Kibria et al., 2019) and type II diabetes (Centers for Disease Control & Prevention, 2017), than non‐Hispanic White individuals. The persistence of this disparity, regardless of improved outcomes in the general population, suggests that traditional risk factors might not fully inform cardiovascular and metabolic disease risk in non‐Hispanic Black individuals (Howard et al., 2018; Leigh et al., 2016; Spanakis & Golden, 2013; Tsao et al., 2022).

Data have shown reduced endothelial nitric oxide (NO)‐dependent vasodilatation in non‐Hispanic Black relative to non‐Hispanic White young adults (Akins et al., 2022; Hurr et al., 2018; Kim et al., 2018; Miller et al., 2021; Ozkor et al., 2014; Patik et al., 2018; Turner et al., 2020; Wolf et al., 2020; Wong et al., 2020). Less is currently known about whether sensory nerve‐mediated vasodilatation differs between non‐Hispanic Black and White young adults. We have demonstrated reduced cutaneous sensory nerve‐mediated vasodilatation in response to local heating in non‐Hispanic Black relative to non‐Hispanic White young adults (Miller et al., 2021; Turner et al., 2020; Wong et al., 2020); however, full expression of sensory nerve‐mediated vasodilatation in response to local heating is dependent on adequate bioavailable NO (Houghton et al., 2006; Minson et al., 2001; Wong & Fieger, 2012; Wong et al., 2020). It is therefore unclear whether there are differences in sensory nerve‐mediated vasodilatation in response to a stimulus that is independent of NO.

Data suggest that the prevalence of neuropathy is higher in the non‐Hispanic Black population even in persons <40 years of age (Eichholz et al., 2017; Hicks et al., 2021; Sun et al., 2022). Whether vascular dysfunction pre‐dates neuropathy (Cameron et al., 2001) or vice versa is not currently clear. Reduced cutaneous sensory nerve‐mediated vasodilatation has also been observed in other populations [e.g., women with a history of a pre‐eclamptic pregnancy (Pyevich et al., 2022)], making assessment of sensory nerve‐mediated vasodilatation clinically relevant.

Cutaneous reactive hyperaemia is mediated largely by cutaneous sensory nerves and endothelium‐derived hyperpolarizing factors (EDHFs) (Larkin & Williams, 1993; Lorenzo & Minson, 2007) but is largely independent of NO (Binggeli et al., 2003; Medow et al., 2007; Wong et al., 2003; Zhao et al., 2004). Cutaneous reactive hyperaemia can be used to assess sensory nerve‐mediated vasodilatation independent of bioavailable NO and might provide a more direct assessment of sensory nerve‐mediated vasodilatation than local heating. The purpose of this study was to test the hypothesis that cutaneous reactive hyperaemia and sensory nerve‐mediated vasodilatation would be lower in non‐Hispanic Black relative to non‐Hispanic White young adults.

## 2 METHODS

### 2.1 Ethical approval

All participants gave both written and verbal informed consent. The Georgia State University Institutional Review Board approved this study (H22572). The study and all protocols conformed to the guidelines set forth in the *Declaration of Helsinki*.

### 2.2 Participants and instrumentation

Participants were 18–40 years of age and self‐identified as non‐Hispanic Black (*n* = 12; six women and six men) or non‐Hispanic White (*n* = 12; seven women and five men). Although there is no consensus on when women should be studied in relationship to their menstrual cycle or contraceptive phase (Stanhewicz & Wong, 2020; Wenner & Stachenfeld, 2020), we tested all women during the low‐hormone phase of the natural menstrual cycle (days 1–5) or the placebo phase of oral contraceptive pill use. All participants were free of cardiovascular and metabolic disease, cancer and nerve damage. Each participant was equipped with an automated blood pressure (BP) cuff (Connex 6300; Welch‐Allyn, Skancateles Falls, NY, USA) on the right arm to obtain BP every 5 min during the protocol. A manual BP cuff was placed on the left arm and was used to occlude arterial inflow and elicit reactive hyperaemia. An index of skin blood flow was measured via laser‐Doppler flowmetry, and a local skin‐heating unit controlled local skin temperature (moorVMS HEAT and LDF2; Moor Instruments, Axminster, UK). Participants were equipped with two laser‐Doppler probes and two local heaters on the dorsal aspect of the left forearm to serve as a control site and as a sensory nerve‐inhibited site. The two sites were separated by at least 3–5 cm.

### 2.3 Protocol

Participants rested in a semi‐recumbent position, with the experimental arm at heart level to control hydrostatic pressure and minimize myogenic responses. One site of the forearm served as a control and a second site was treated with over‐the‐counter topical 4% lignocaine (Aspercreme with 4% lidocaine; Chattem, Chattanooga, TN, USA), which was applied to an area of skin ∼5 cm^2^ to inhibit sensory nerve function and covered with an adhesive dressing (Tegaderm; 3M, St. Paul, MN, USA) for 90 min. After 90 min, lignocaine was wiped off the skin and a second application was made for 30 min. After the second application of lignocaine, sensory nerve function was assessed by lightly scratching the skin with the sharp end of surgical scissors and a paperclip. We recently used lignocaine to inhibit sensory nerve function (Wong et al., 2020). After two applications of lignocaine, all participants indicated that they could no longer feel the sharp end of the surgical scissors or paper clip, indicating effective inhibition of sensory nerve function. Inhibition of sensory nerve function was confirmed at the end of the study using the same procedures.

Baseline data were measured for 10 min with local skin heaters at 33°C. After baseline measurements, three arterial occlusions and reactive hyperaemic responses were measured. For each bout, the manual BP cuff was rapidly inflated to suprasystolic pressure (250 mmHg) to occlude arterial inflow for 5 min. The pressure in the manual cuff was then rapidly released to elicit reactive hyperaemia. At least 10–15 min of recovery were allowed between bouts (Wong et al., 2003). After the third reactive hyperaemia, the two sites were heated to 43°C at a rate of 0.1°C s^−1^, which was shown to induce maximal cutaneous vasodilatation (Holowatz et al., 2005; McNamara et al., 2014; Minson et al., 2001; Wong & Fieger, 2010).

### 2.4 Data analysis

All data were sampled at 40 Hz (PowerLab 16/35; ADInstruments, Colorado Springs, CO, USA) and stored and analysed on a laboratory computer. Mean arterial pressure (MAP) was calculated as one‐third pulse pressure plus diastolic pressure, and cutaneous vascular conductance (CVC) was calculated as laser‐Doppler flux divided by MAP and normalized to maximal vasodilatation percent maximal cutaneous vascular conductance (%CVC_max_). Baseline values were averaged over a 3 min period preceding each arterial occlusion.

Peak reactive hyperaemia was defined as the largest increase in skin blood flow after release of the manual BP cuff and expressed as percent maximal cutaneous vascular conductance. Total reactive hyperaemia was calculated as:

$$\begin{array}{ccc} \mathrm{Total}\;\mathrm{reactive}\;\mathrm{hyperaemia}\; & = & \;\mathrm{AUC}-\left(\mathrm{baseline}\;\right.\\ & & \left.\times \;\;\mathrm{duration}\;\mathrm{of}\;\mathrm{reactive}\;\mathrm{hyperaemia}\right), \end{array}$$

where AUC is the area under the reactive hyperaemia curve, baseline is baseline skin blood flow preceding arterial occlusion, and duration of reactive hyperaemia is the time it took for reactive hyperaemia to return to baseline. This equation takes into account baseline differences between trials and participants and only includes the increase in skin blood flow above baseline. Data for total reactive hyperaemia are expressed as percent maximal cutaneous vascular conductance x seconds (Wong et al., 2003). We previously reported a large degree of variability in the cutaneous reactive hyperaemic response (Wong et al., 2003) and used the two most consistent reactive hyperaemia trials for final statistical analysis (Wong et al., 2003). The contribution of sensory nerves to both peak and total reactive hyperaemia was calculated as:

$$\%\mathrm{Sensory}\;\mathrm{nerves}=\left[\frac{\mathrm{Control}-\mathrm{Lignocaine}}{\mathrm{Control}}\right]\;\times \;100.$$

### 2.5 Statistical analysis

A priori power analysis was calculated for peak and total hyperaemia using preliminary data from our laboratory. For both phases of reactive hyperaemia, sample size was calculated using means and (SD) with an α level of 0.05 and power of 0.8. Peak reactive hyperaemia pilot data [non‐Hispanic Black, 51 (12)%CVC_max_; non‐Hispanic White, 68 (13)%CVC_max_] yielded a sample size of 10 participants per group. Total reactive hyperaemia pilot data [non‐Hispanic Black, 2750 (1010)%CVC_max_ s; non‐Hispanic White, 4242 (1400)%CVC_max_ s] yielded a sample size of 12 participants per group. For our final sample size, we used the higher sample size calculated for total reactive hyperaemia.

Statistical analyses were performed using SPSS v.28 (IBM Corporation, Armonk, NY, USA). Levene's test of equality of variances was performed. Statistical significance was set a priori at *P* ≤ 0.05. All data are presented as the mean (SD). Peak and total reactive hyperaemia, baseline and maximal responses were compared using a two‐way ANOVA with between‐subject factors of racial identity (non‐Hispanic Black and White) and within‐subject factors of treatment (control and lignocaine). Calculated sensory nerve contributions to peak and total reactive hyperaemia were compared using Student's unpaired *t*‐tests.

## 3 RESULTS

Participants were well matched for all anthropometric and haemodynamic parameters (Table 1).

**TABLE 1:** Anthropometric and haemodynamic descriptive statistics for all participants (*n* = 12 participants in each group).

Parameter	Non‐Hispanic Black young adults (*n* = 12)	Non‐Hispanic White young adults (*n* = 12)
Women/men (*n*)	6/6	7/5
Age (years)	22 (3)	24 (2)
Height (m)	1.72 (0.13)	1.66 (0.12)
Mass (kg)	75.0 (13.7)	70.0 (10.2)
BMI (kg m^−2^)	25.2 (2.7)	25.5 (2.4)
Systolic BP (mmHg)	115 (6)	113 (6)
Diastolic BP (mmHg)	72 (4)	70 (6)
Mean arterial pressure (mmHg)	86 (4)	84 (5)
Heart rate (beats min^−1^)	62 (7)	62 (8)

*Note*: Data are shown as the mean (SD).

Abbreviations: BMI, body mass index; BP, blood pressure.

**TABLE 2:** Baseline and maximal skin blood flow data.

	Non‐Hispanic Black young adults (*n* = 12)		Non‐Hispanic White young adults (*n* = 12)	
Blood flow	Control	Lignocaine	Control	Lignocaine
Baseline (%CVC_max_)	14 (4)	15 (6)	16 (5)	16 (7)
Maximal CVC (flux mmHg^−1^)	2.31 (0.72)	2.27 (0.57)	2.33 (1.07)	2.23 (1.09)

*Note*: Data are the mean (SD). Data were compared using a two‐way ANOVA. There were no significant main or interaction effects for either baseline %CVC_max_ or maximal CVC. CVC, cutaneous vascular conductance; %CVC_max_, percent maximal cutaneous vascular conductance. See main text for *P*‐values.

### 3.1 Baseline and maximal data (Table 2)

There were no significant main or interaction effects for baseline (treatment *P* = 0.39; racial identity *P* = 0.78; interaction *P* = 0.78) or maximal CVC (treatment *P* = 0.79; racial identity *P* = 0.97; interaction *P* = 0.91).

### 3.2 Peak reactive hyperaemia (Figure 1)

There was a main effect of racial identity and treatment (*P* < 0.001 for both), but there was no interaction effect (*P* = 0.895) for peak reactive hyperaemia. Peak reactive hyperaemia was reduced in non‐Hispanic Black relative to non‐Hispanic White participants at both the control site [non‐Hispanic Black, 60 (14)%CVC_max_; non‐Hispanic White, 78 (14)%CVC_max_] and the lignocaine site [non‐Hispanic Black, 44 (14)%CVC_max_; non‐Hispanic White, 64 (15)%CVC_max_). There was no difference in the calculated contribution of sensory nerves to peak reactive hyperaemia between non‐Hispanic Black [25 (15)%] and non‐Hispanic White [18 (13)%; *P* = 0.247] young adults.

**FIGURE 1:**
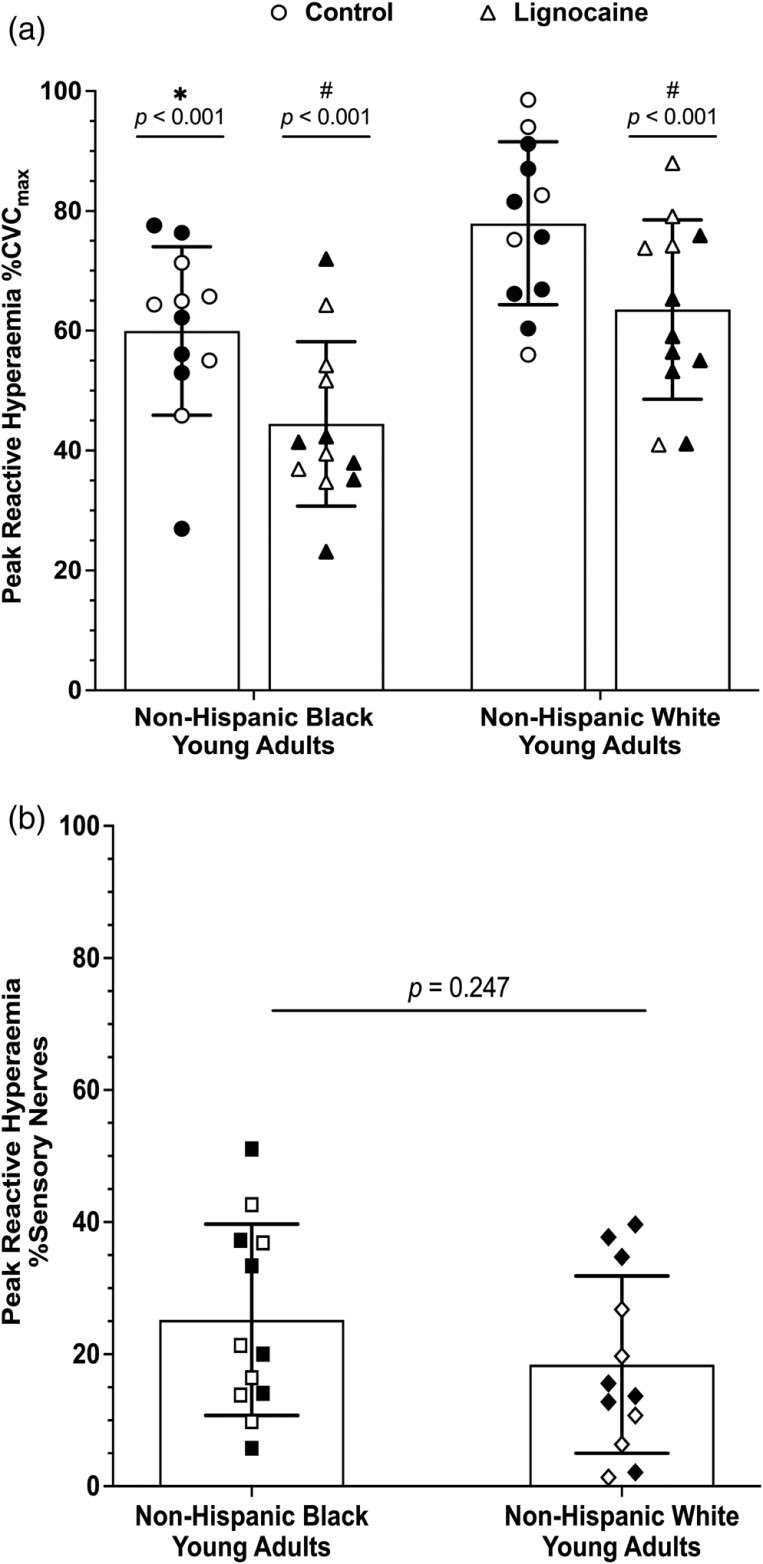
Peak reactive hyperaemia. (a) Peak reactive hyperaemia in non‐Hispanic Black (*n* = 12; six women and six men) and White (*n* = 12; seven women and five men) participants at control (circles) and lignocaine (triangles) sites. Filled symbols are data from women; open symbols are data from men. ^*^ *P* < 0.001 control versus non‐Hispanic White participants. ^#^ *P* < 0.001 lignocaine versus respective control sites. Data were analysed via two‐way ANOVA. (b) Calculated percentage sensory nerve contribution to peak reactive hyperaemia in non‐Hispanic Black (squares) and non‐Hispanic White (diamonds) young adults. There was no difference in percentage sensory nerve contribution between groups. Data were analysed by Student's unpaired *t*‐test.

### 3.3 Total reactive hyperaemia (Figure 2)

There was a significant interaction effect (*P* = 0.020) for total reactive hyperaemia. Total reactive hyperaemia was lower in non‐Hispanic Black relative to non‐Hispanic White participants at both the control site [non‐Hispanic Black, 4085 (955)%CVC_max_ s; non‐Hispanic White, 6820 (1179)%CVC_max_ s; *P* < 0.001] and the lignocaine site [non‐Hispanic Black, 2127 (639)%CVC_max_ s; non‐Hispanic White, 3573 (712)%CVC_max_ s; *P* < 0.001]. Total reactive hyperaemia was also significantly reduced at lignocaine compared with control sites within each group (*P* < 0.001 for both groups). There was no significant difference in the calculated contribution of sensory nerves to total reactive hyperaemia between non‐Hispanic Black [48 (10)%] and non‐Hispanic White [47 (10)%, *P* = 0.853] participants.

**FIGURE 2:**
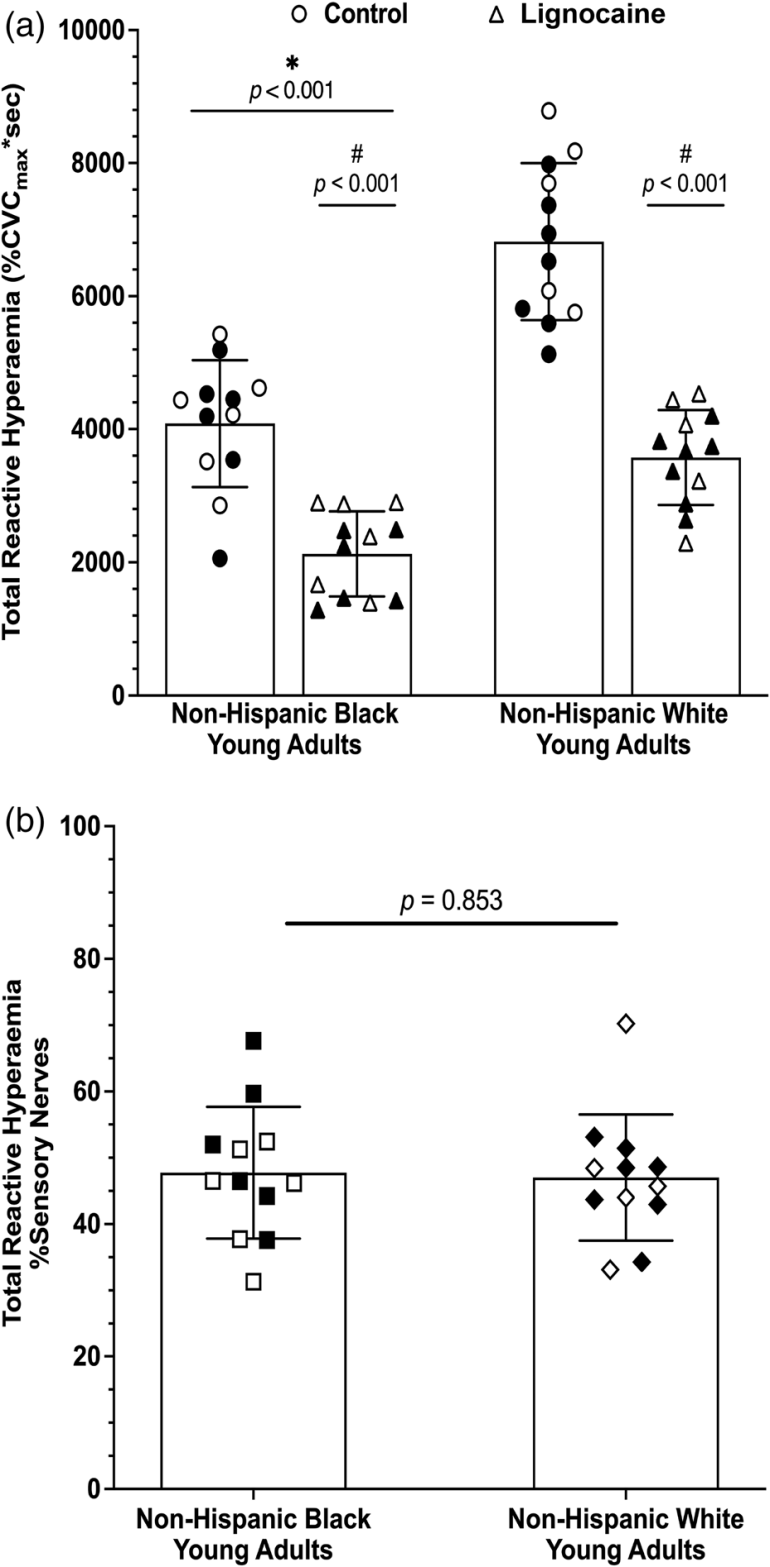
Total reactive hyperaemic response. (a) Total reactive hyperaemia in non‐Hispanic Black (*n* = 12; six women and six men) and White (*n* = 12; seven women and five men) participants at control (circles) and lignocaine (triangles) sites. Filled symbols are data from women; open symbols are data from men. ^*^ *P* < 0.001 both control and lignocaine sites versus non‐Hispanic White participants. ^#^ *P* < 0.001 lignocaine versus respective control sites. Data were analysed via two‐way ANOVA. (b) Calculated percentage sensory nerve contribution to total reactive hyperaemia in non‐Hispanic Black (squares) and non‐Hispanic White (diamonds) young adults. There was no difference in percentage sensory nerve contribution between groups. Data were analysed by Student's unpaired *t*‐test.

## 4 DISCUSSION

In agreement with our hypothesis, we found that both peak and total cutaneous reactive hyperaemia were lower at control sites in non‐Hispanic Black relative to non‐Hispanic White young adults. In contrast to our hypothesis, the calculated sensory nerve contribution to both peak and total cutaneous reactive hyperaemia was similar between groups.

Although the calculated sensory nerve contribution to both peak and total reactive hyperaemia was similar in both groups, the overall magnitude of reactive hyperaemia was lower in non‐Hispanic Black young adults. Thus, sensory nerves make a similar contribution to a lower overall response in non‐Hispanic Black young adults. This suggests that mechanisms other than sensory nerves are responsible for the difference in magnitude of both peak and total reactive hyperaemia between non‐Hispanic Black and White participants. It is unlikely that NO is responsible given that we have previously shown that cutaneous reactive hyperaemia is unaltered by NO synthase inhibition, and there is no appreciable increase in interstitial NO levels (Wong et al., 2003; Zhao et al., 2004). Previous data suggest that cutaneous reactive hyperaemia is largely mediated by both cutaneous sensory nerves and EDHFs (Larkin & Williams, 1993; Lorenzo & Minson, 2007). Thus, EDHF‐dependent mechanisms might be lower in non‐Hispanic Black relative to non‐Hispanic White young adults and might be responsible for the lower magnitude of both peak and total reactive hyperaemia; however, previous data suggest that EDHF‐dependent mechanisms might be preserved in healthy African American adults (Ozkor et al., 2014). The role of EDHF mechanisms in microvascular function in healthy non‐Hispanic Black adults remains unknown.

It is possible that myogenic mechanisms underlie the differences in magnitude of cutaneous reactive hyperaemia between non‐Hispanic Black and White participants. Myogenic reactions contribute to reactive hyperaemia in the skin and muscle vasculature (Blair et al., 1959; Patterson, 1956; Rogers & Sheriff, 2005), and myogenic mechanisms can have a profound effect on vascular conductance (Wong & Sheriff, 2008). Alterations in downstream pressure during arterial occlusion might persist during the early phases of reperfusion and contribute to differences in reactive hyperaemia. Although this has not been assessed experimentally in humans, differences in myogenic responses in isolated resistance arterioles from non‐Hispanic Black and White adults have been documented (Sabbahi et al., 2021).

Sensory nerves were previously shown to contribute ∼50% to peak and ∼70% to total reactive hyperaemia (Lorenzo & Minson, 2007), whereas our data suggest that sensory nerves only contribute ∼20–25% to the peak and ∼50% to total reactive hyperaemia. It is unclear why we observed such stark differences in the sensory nerve contribution to cutaneous reactive hyperaemia. It is possible that differences in the anaesthetic used in each study [eutectic mixture of local anaesthetic in the Lorenzo & Minson (2007) and lignocaine in the present study] contributed to the observed differences. Use of 4% lignocaine effectively inhibits menthol‐induced vasodilatation (Craighead & Alexander, 2017), suggesting that eutectic mixture of local anaesthetic and lignocaine are equally effective at inhibiting sensory nerve‐mediated vasodilatation. The magnitude of responses observed in the present study was similar to what we reported previously (Wong et al., 2003), and in both our studies, the average responses were larger than those observed by Lorenzo and Minson (2007). It is therefore possible that topical anaesthetic was not able to inhibit the more robust reactive hyperaemic responses maximally.

### 4.1 Limitations

First, we did not use microdialysis to inhibit EDHFs. The aim of this study was to assess sensory nerve‐mediated cutaneous vasodilatation, independent of bioavailable NO, in non‐Hispanic Black and White young adults, and therefore, we did not inhibit EDHFs. We also sought to determine whether reactive hyperaemia combined with topical anaesthetic could be used as a simple, non‐invasive method to assess sensory nerve function.

Second, we did not assess social determinants of health, which are known to contribute to health disparities. Recent data showed that socioeconomic status was lower in young, healthy African Americans but was not correlated with cutaneous vasodilatation (Wolf et al., 2020). Although all our participants were college students, various social determinants of health could have contributed to the observed physiological responses. A much larger sample size would be required to observe statistical differences in survey/questionnaire data, and a larger cohort prospective study would be better suited to address this.

Third, we collected venous blood samples but were unable to obtain samples from all participants (missing samples for two or three participants per group), and we had technical problems with the analysis for about half of the samples (three or four per group). As such, we do not have a complete blood profile of the entire cohort, and it is possible that some participants had abnormal glucose and/or lipid values.

Fourth, some data have shown that lignocaine itself can either cause vasoconstriction or blunt vasodilatation (Gherardini et al., 1996; Jernbeck & Samuelson, 1993; Wadström & Gerdin, 1991), and it is possible that lignocaine exerted non‐specific effects on the vasculature. We observed no difference in baseline values between control and lignocaine sites, which would suggest that lignocaine did not elicit vasoconstriction; however, we cannot rule out this possibility completely.

Finally, it is possible that heating to 43°C was not sufficient to elicit a true maximum, particularly at the lignocaine site. If this were the case, then our data would be an overestimation of reactive hyperaemia, but this would not necessarily negate the overall findings of the study. The lack of a difference between maximal CVC at control and lignocaine sites would suggest that the response to heating to 43°C was not affected by lignocaine.

## 5 CONCLUSIONS

We show that both peak and total cutaneous reactive hyperaemia are lower in non‐Hispanic Black relative to non‐Hispanic White young adults. However, the contribution of sensory nerves to both peak and total reactive hyperaemia is similar between groups. These data suggest that mechanisms other than sensory nerves are responsible for lower cutaneous reactive hyperaemia in non‐Hispanic Black young adults. Future studies aimed at investigating these potential mechanisms, including EDHFs and myogenic responses, are warranted.

## AUTHOR CONTRIBUTIONS

All data were collected in the Department of Kinesiology & Health at Georgia State University. Casey Turner was responsible for data collection and drafting and editing the manuscript. Demetria Walker was responsible for data collection and drafting and editing the manuscript. Brett Wong was responsible for experimental and conceptual design, data analysis, and editing all drafts of the manuscript. All authors approved the final version of the manuscript and agree to be accountable for all aspects of the work in ensuring that questions related to the accuracy or integrity of any part of the work are appropriately investigated and resolved. All persons designated as authors qualify for authorship, and all those who qualify for authorship are listed.

## CONFLICT OF INTEREST

None declared.

## Supporting information

Statistical Summary Document

## Data Availability

The data that support the findings of this study are available from the corresponding author upon reasonable request.
